# 

**Published:** 1901-09

**Authors:** 


*The following donations have been received since the publication of the List in June:*

*August 31st, 1901.*

| | |
|---|---|
| L. M. Griffiths (1) ... ... ... ... ... ... ... | 4 volumes. |
| London Homœopathic Hospital (2) ... ... ... | 5 ,, |
| Medical Library, McGill University (3) ... ... | 4 ,, |
| George Parker, M.D. (4)... ... ... ... ... ... | 3 ,, |
| R. Shingleton Smith, M.D. ... ... ... ... ... | 1 volume. |
| Miss C. M. Swayne (5) ... ... ... ... ... ... | 32 volumes. |

### FORTY-FIRST LIST OF BOOKS.

The titles of books mentioned in previous lists are not repeated.

The figures in brackets refer to the figures after the names of the donors, and show by whom the volumes were presented. The books to which no such figures are attached have either been bought from the Library Fund or received through the *Journal*.

**Allchin, W. H.** [Ed.] *A Manual of Medicine.* Vol. III. ... ... ... 1901

**Bland-Sutton,** *see* **Sutton.**

**Burghard, W. W. Cheyne and F. F.** *A Manual of Surgical Treatment.* Part V. ... ... ... ... ... ... ... ... ... 1901

**Butlin, H. T.** ... *The Operative Surgery of Malignant Disease* 2nd Ed. 1900

*Cheltenham, A Guide to* (Edited by E. T. Wilson and J. Sawyer) (1) 1901

**Cheyne and F. F. Burghard, W. W.** *A Manual of Surgical Treatment.* Part V. ... ... ... ... ... ... ... ... ... 1901

**Coulliaux, L.** ... *Igiene della Bocca e dei Denti* ... ... ... ... ... 1901

**Cullen, T. S.** ... *Cancer of the Uterus* ... ... ... ... ... .. ... 1900

**Engelmann [—]** ... *Results of the Open-Air Treatment of Consumption* ... 1901
**Fenwick, E. H.** ... *Tumours of the Urinary Bladder* ... ... ... ... ... ... 1901
**Fox, E. L.** ... ... *The Pathological Anatomy of the Nervous Centres* (5) 1874
**Gardini, R. Stecchi e A.** *Chirurgia Operatoria* ... ... ... ... ... ... ... 1901
**Gibson, G. A.** [Ed.] *Text-Book of Medicine.* 2 vols. ... ... ... ... ... 1901
**Glasson, C. J.** ... *Motherhood* ... ... ... ... ... ... ... ... ... ... 1901
**Goodsall and W. E. Miles, D. H.** *Diseases of the Anus and Rectum.* Part I. ... ... ... ... ... ... ... ... ... ... 1900
**Hewitt, F. W.** ... *Anæsthetics and their Administration* ... ... ... ... ... 1901
**Hime, M. C.** ... *Schoolboys' Special Immorality* ... ... ... 3rd Ed. 1901
**Hutchison, R.** ... *Food and the Principles of Dietetics* ... ... ... ... ... 1901
**Lane, W. A.** ... *Fractures, Cleft Palate, etc.* ... ... ... ... 2nd Ed. [1900]
**McCallin, W.** ... *Medical Jurisprudence* ... ... ... ... ... ... ... ... 1901
**Majnoni, R.** ... *Massaggio* ... ... ... ... ... ... ... ... ... ... ... 1901
**Makins, G. H.** ... *Surgical Experiences in South Africa,* 1899—1900 ... 1901
*Medical and Surgical History of the British Army which served in the War against Russia,* 1854-55-56. 2 vols. ... ... (2) 1858
**Miles, D. H. Goodsall and W. E.** *Diseases of the Anus and Rectum.* Part I. ... ... ... ... ... ... ... ... ... ... 1900
**Morgan, J.** ... *Diseases of the Eye* ... ... ... ... ... ... ... ... (5) 1839
**Morris, H.** ... ... *Surgical Diseases of the Kidney and Ureter.* 2 vols. 1901
**Pieraccini, A.** ... *L'Assistenza dei Pazzi* .. ... ... ... ... ... ... ... 1901
**Phelps, C.** ... ... *Traumatic Injuries of the Brain and its Membranes.* 2nd Ed. 1900
**Phillips, J.** ... *Diseases of Women* ... ... ... ... ... ... 3rd Ed. 1901
**Poland, J.** ... ... *A Retrospect of Surgery during the Past Century* ... 1901
**Spurrell, H. G. F.** *The Commonwealth of Cells* ... ... ... ... ... ... ... 1901
**Stecchi e A. Gardini, R.** *Chirurgia Operatoria* ... ... ... ... ... ... 1901
**Sutton, J. B.** ... *Tumours, Innocent and Malignant*... New [2nd] Ed. 1901
**Thomson, G. S. and J.** *A Treatise on Plague* ... ... ... ... ... ... ... 1901
**Tilley, H.** ... ... *Purulent Nasal Discharges* ... ... ... ... ... ... .. 1901
*Tuberculosis, Descriptive Catalogue of the Museum of the British Congress on, London,* 1901 ... ... ... ... ... ... ... ... ... 1901
*Tuberculosis and other Diseases insured by the Insurance Institutions* [*etc.*] *of German Invalidity Insurance, for the years* 1897—1900, *Statistics of Medical Treatment of Persons suffering from* ... ... ... ... ... ... ... ... ... 1901
**Tweedie, A.** ... *Clinical Illustrations of Fever* ... ... ... ... (1) 1830
**Waldo, F. J.** ... *Golden Rules of Hygiene* ... ... ... ... ... ... [1901]
**Wilson, E.** ... ... *The Anatomist's Vade Mecum* ... ... (5) 4th Ed. 1847
**Wood, W.** ... ... *Insanity and the Lunacy Law* ... ... ... ... (1) 1879

### TRANSACTIONS, REPORTS, JOURNALS, &c.

Allgemeine Medicinische Central-Zeitung ... ... ... ... ... ... (4) 1889-91
American Journal of Ophthalmology, The ... ... ... ... Vol. XVII. 1900
American Journal of the Medical Sciences, The ... ... Vol. CXX. 1900
American Laryngological Association, Transactions of the ... ... ... 1901
American Practitioner and News, The... ... ... Vols. XXIX., XXX. 1900
Annales de la Société Belge de Chirurgie ... ... ... ... Tome VIII. 1900
Annali di Ostetricia e Ginecologia ... ... ... ... ... Vol. XXII. 1900
Annual and Analytical Cyclopædia of Practical Medicine ... Vol. VI. 1901

| | | |
|---|---|---|
| Archives of the Roentgen Ray ... .. ... ... ... ... ... | Vol. IV. | 1900 |
| Australasian Medical Gazette, The ... ... ... ... ... | Vol. XIX. | 1900 |
| Bibliographia Medica (Index Medicus)... ... ... ... ... ... | Tome I. | 1900 |
| British Gynæcological Journal, The ... ... ... ... ... | Vol. XVI. | 1900 |
| British Medical Journal, The ... ... ... ... ... ... ... ... | Vol. I. for | 1901 |
| Bulletin de l'Académie royale de Médecine de Belgique, | IVe Sér., Tome XIV. | 1900 |
| Canada Medical and Surgical Journal ... ... ... | (3) Vols. I.—IV. | 1873-76 |
| Congrès (XIIIe) international de Médecine, Paris, 1900. — Comptes Rendus ... ... ... | [Vols. IV., VI., IX., X., XII., XIV., XVII. | 1901] |
| Congrès (XIIIe) international de Médecine, Paris, 1900.—Organisation, Assemblées générales, [etc.] ... ... ... ... ... ... ... ... | | 1901 |
| Edinburgh Medical Journal, The ... ... ... ... ... | N.S., Vol. IX. | 1901 |
| Hospital, The ... ... ... ... ... ... ... ... ... ... ... | Vol. XXIX. | 1901 |
| Hospitals— | | |
| Glasgow Hospital Reports ... ... ... ... ... ... ... | Vol. III. | 1901 |
| Johns Hopkins Hospital Reports, The | Vols. VIII., 1900; X., Nos. 1–2 | 1901 |
| Saint Thomas's Hospital Reports ... ... | N.S., Vol. XXVIII. | 1901 |
| Hospitals and Charities, Burdett's... ... ... ... ... ... ... ... ... ... | | 1901 |
| Intercolonial Medical Journal of Australasia ... ... ... ... | Vol. V. | 1900 |
| Journal de Neurologie ... ... ... ... ... ... ... ... ... | Tome V. | 1900 |
| Journal of Balneology and Climatology, The ... ... ... ... | Vol. IV. | 1900 |
| Journal of the American Medical Association, The | Vols. XXXIV., XXXVI. | 1900-01 |
| Lancet, The... ... ... ... ... ... ... ... ... ... ... ... | Vol. I. for | 1901 |
| Library, The ... ... ... ... ... ... ... .. ... ... | N.S., Vol. I. | 1900 |
| Liverpool Medico-Chirurgical Journal, The .. ... ... ... | Vol. XX. | 1900 |
| London Medical Review, The... ... ... ... ... ... | (2) Vols. II., III. | 1862-63 |
| Medical Chronicle, The ... ... ... ... ... ... ... | 3rd Ser., Vol. IV. | 1900-01 |
| Medical Times, The ... ... ... ... ... ... ... ... ... | (2) Vol. XVI. | 1847 |
| Northumberland and Durham Medical Journal, The, 1900 ... ... ... | | 1901 |
| Obstetrical Transactions ... ... ... ... ... ... ... ... | Vol. XLII. | 1901 |
| Occidental Medical Times ... ... ... ... ... ... ... ... | Vol. XIV. | 1900 |
| Philadelphia County Medical Society, Proceedings of the | Vol. XXI. | 1900 |
| Post-Graduate, The ... ... ... ... ... ... ... ... ... ... | Vol. XV. | 1900 |
| Progressive Medicine ... ... ... ... ... ... ... ... | Vols. I., II. | 1901 |
| Retrospect of Medicine, Braithwaite's... ... ... ... | Vol. CXXIII. | 1901 |
| Revue générale d'Ophtalmologie .. ... ... ... ... | Vol. XIX. | 1900 |

---

We are indebted to the Honorary Librarian, Mr. L. M. Griffiths, for the following note on

### MEDICAL LIBRARIES.

At the dedication of the new building of the Boston Medical Library many interesting speeches were made. From these I should like to quote much, but space allows only a few extracts. The librarian, Dr. James R. Chadwick, laid stress on the importance of completing the files of all the important periodicals. Dr. William Osler said that for the general practitioner a well-used library is one of the few correctives of the premature senility which is apt to overtake him. Self-centred, self-taught, he leads a solitary life, and unless his everyday experience is controlled by careful reading or by the attrition of a medical society it soon ceases to be of the slightest value and becomes a mere accretion of isolated facts, without correlation. It is astonishing with how little reading a doctor can practise medicine, but it is not astonishing how badly he may do it. More bibliomaniacs are needed. Along two lines their work is valuable. By the historical method alone can many problems in medicine be approached profitably. For example, the student who dates his knowledge of tuberculosis from Koch may have a very correct, but he has a very incomplete appreciation of the subject. Within a quarter of a century our libraries will have certain alcoves devoted to the historical consideration of the great diseases, which will give to the student that mental perspective which is so valuable an equipment in life. Dr. J. S. Billings pointed out that the funds for conducting a library, medical or other, are always insufficient, and the librarian, or library committee, must therefore exclude from the purchase-lists many works which might be welcome additions if obtainable from other sources. The department of first importance in a medical library is that which contains its files of periodicals, not only because they contain the original records from which text-books and monographs are made up, but they represent the feelings, views, and wants of the great mass of the profession, and are the great sources for the medical history of the nineteenth century. Medicine is now the most cosmopolitan and international of all the arts and professions, and that is largely due to its periodicals. Moreover, its periodical literature is now more accessible than that of any other profession because of the indexes upon which Dr. Holmes so much insisted. Dr. Weir Mitchell, who was unable to be present, sent a letter in which he said: "I see as I grow old, or may I say older, how few are the young medical men whose tastes are scholarly, and who, like Holmes and our own lamented Da Costa, are familiar with the fathers and find pleasure in the old books—the quaint books, the sense and the vagaries of the past. Without a great library few can afford these intellectual playgrounds, or, indeed, acquire the material for such indulgence. . . . I often remember with regret the great waste of time in my younger days when there were no great libraries and when John Billings had not indexed the medical thought of all the centuries. The enormous labor then involved in any mere literary research as to facts no one can imagine to-day. A great library of medicine is truly a labor-saving machine, and . . . well managed practically lengthens life by saving time." The *Boston Medical and Surgical Journal*, from which these extracts are taken, says: "The fact must not for a moment be lost sight of that much more remains to be done; that with new privileges come new responsibilities of a very definite sort. The library must be supported as never before; it must continually have new books, and widen its scope of usefulness in every possible direction. To this end a renewed interest must be taken in its affairs by every one, whether remotely or intimately connected with it. . . . It behooves us to recognize our new responsibilities and by all ways in our power to enhance the usefulness of the library."

*Mutato nomine de te fabula narratur.* Nearly all that was so well said about the excellent institution in Boston may be said of the Bristol Medical Library, and if those who have its welfare at heart would form a permanent endowment fund or establish it on a firmer basis by annual subscriptions, large or small, or by substantial single donations, it might be made immeasurably more useful than it even is at present and would conduce to the practical and permanent benefit of the profession.

Our library is becoming more and more recognised by doctors outside Bristol as a useful institution, and it is not too much to hope that in time it will be considered the medical centre of a large and influential district. With increased facilities of travel it can be resorted to by many living outside the city, who would doubtless bear their share in maklng it thoroughly representative of the best interests of the profession. In the words of a circular recently sent to doctors in and about Boston, we may say that our library "offers a convenient and pleasant place for friendly meeting; the information which will some day be indispensable to you is here ready to your hand; those of your sons who follow in your steps will find here the inspiration essential to their success."

### Local Medical Notes.

### BRISTOL.

The Bristol Medical School.—Mr. Newman Neild, M.B., Ch. B. Vict., has been appointed Assistant-Pathologist to the Bristol General Hospital; Mr. S. V. Stock, M.B., B.S. Lond., has been appointed Senior Resident Medical Officer at the Manchester Royal Infirmary. In the last Intermediate M.B. Examination of the Cambridge University, Messrs. F. Leighton, B.A., and L. Shingleton Smith, B.A., were successful candidates. The following Scholarships and Prizes have been recently awarded: the Committee Silver Medal to Mr. G. D. Edwards; the Augustin Prichard Prize to Mr. C. F. Walters; the Clarke Scholarship to Mr. A. R. Short; the Marshall Prize to Mr. A. R. Short; the Sanders Scholarship to Mr. J. E. Sparks, 1st, and Mr. C. Corfield, 2nd.

